# Mapping of incisional reentrant tachycardia: Lessons learned

**DOI:** 10.1016/j.ipej.2026.06.005

**Published:** 2026-06-10

**Authors:** Shankar Baskar, Jeremy P. Moore

**Affiliations:** a The Heart Institute, Cincinnati Children's Hospital Medical Center, 3333 Burnet Avenue, Cincinnati, OH, 45229, USA; b University of California Los Angeles (UCLA) Cardiac Arrhythmia Center, UCLA Health System, Los Angeles, CA, USA; c Ahmanson/UCLA Adult Congenital Heart Disease Center, Department of Medicine, Division of Cardiology, Los Angeles, CA, USA

## 1 Introduction

Adults with congenital heart disease (ACHD) develop atrial arrhythmia progressively with age [1]. As this patient population continues to grow, the associated arrhythmia burden will continue to challenge existing healthcare systems such that increasing specialized expertise is required [2]. Accordingly, training pathways and center requirements have been developed to provide guidance for this growing need [3,4]. This review focuses on the most common arrhythmia problems encountered by electrophysiologists treating patients with ACHD; that is, atrial arrhythmia. A proposed strategy for catheter ablation for patients with complex congenital substrates and intra-atrial reentrant tachycardia (IART) is provided, along with illustrative examples highlighting the key challenges in this population.

### 1.1 Scope of the problem

Arrhythmias represent one of the most significant long-term sources of health care utilization in ACHD patients. More than 50% of patients with severe ACHD will go onto develop atrial arrhythmias by age 65 [1]. Although a broad spectrum of rhythm disorders including focal atrial tachycardia, atrial fibrillation (AF), and ventricular tachycardia can occur in this population, IART related to postoperative atrial incisional scars and atrial remodeling, remains the predominant arrhythmia mechanism [5]. Because patients with ACHD often have fragile hemodynamics, IART may be poorly tolerated, leading to rapid clinical deterioration, heart failure exacerbation, or even life-threatening instability. As a result, rhythm control is typically a central therapeutic objective rather than a purely symptomatic strategy [6]. Unfortunately, medical therapy is often limited by modest efficacy and potential adverse reactions, making catheter ablation the cornerstone of management for many patients. As the ACHD population ages, AF is increasingly prevalent in this population, and emerging data suggest that earlier identification and aggressive treatment of atrial arrhythmia substrates may help reduce progressive atrial remodeling and potentially delay or mitigate later AF development [7]. This epidemiology highlights the importance of proactive electrophysiologic surveillance and specialized rhythm management in ACHD.

## 2 Case workflow

### 2.1 Pre-procedural preparation

Pre-procedural preparation is of paramount importance for any intervention in post-operative ACHD patients, and this is specifically applicable to electrophysiology studies (EPS).

### 2.2 Arrhythmia

**Fig. 1:**
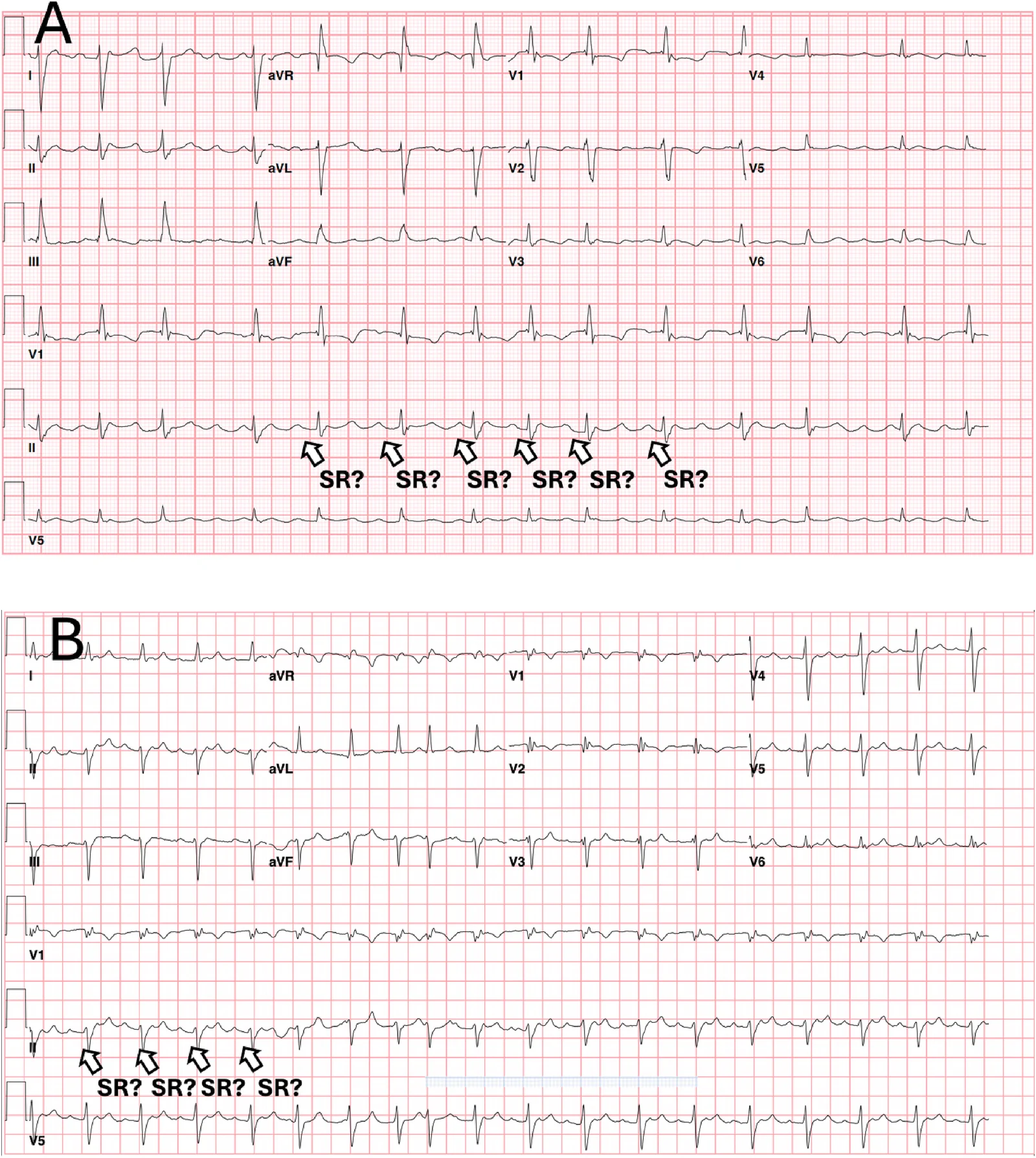
Electrocardiographic interpretation after the Fontan operation. Examples of patients with intra-atrial reentrant tachycardia mistakenly diagnosed with sinus rhythm (A) and sinus tachycardia (B) at initial cardiac evaluation. Broad and variable p wave morphology often masquerades as normal rhythm in this population, requiring increased vigilance. Abbreviation: SR, sinus rhythm.

Documentation of the arrhythmia of concern is imperative Although the present article focuses on incisional re-entry, the operator should have an open mind when approaching these arrhythmias since other forms (atrioventricular reentrant tachycardia, AV node reentrant tachycardia, focal atrial tachycardia) can co-exist and masquerade as an incisional re-entry. Attention should focus on tachycardia cycle length (TCL), AV relationship, initiation/termination and QRS morphology changes. Since ACHD patients with multiple scars can have multiple re-entrant circuits, a general idea of the predominant tachycardia is important. The TCL can often be used in this regard and can be used to target the primary tachycardia in patients in whom multiple atrial arrhythmias are initiated. Macro-re-entrant atrial tachycardia in patients with significant scar burden might have an iso-electric period between P waves on the ECG mimicking a focal atrial tachycardia. Conversely, Fontan patients often have slow atrial tachycardia with low amplitude and/or broad p waves which can confound electrocardiographic interpretation (Fig. 1). AVRT via twin AV nodes is a possibility in patients with CCTGA and isomerism or heterotaxy syndromes and can often be suspected when there is a discrete change in QRS complex. Lastly, the etiology of atrial fibrillation (AF) in this population might be quite different from patients with a structurally normal heart. It is even possible to encounter an irregular exit of a rapid focal atrial tachycardia mimicking AF in patients with multiple atrial scars such as post-Fontan.

### 2.3 Anatomy

**Fig. 2:**
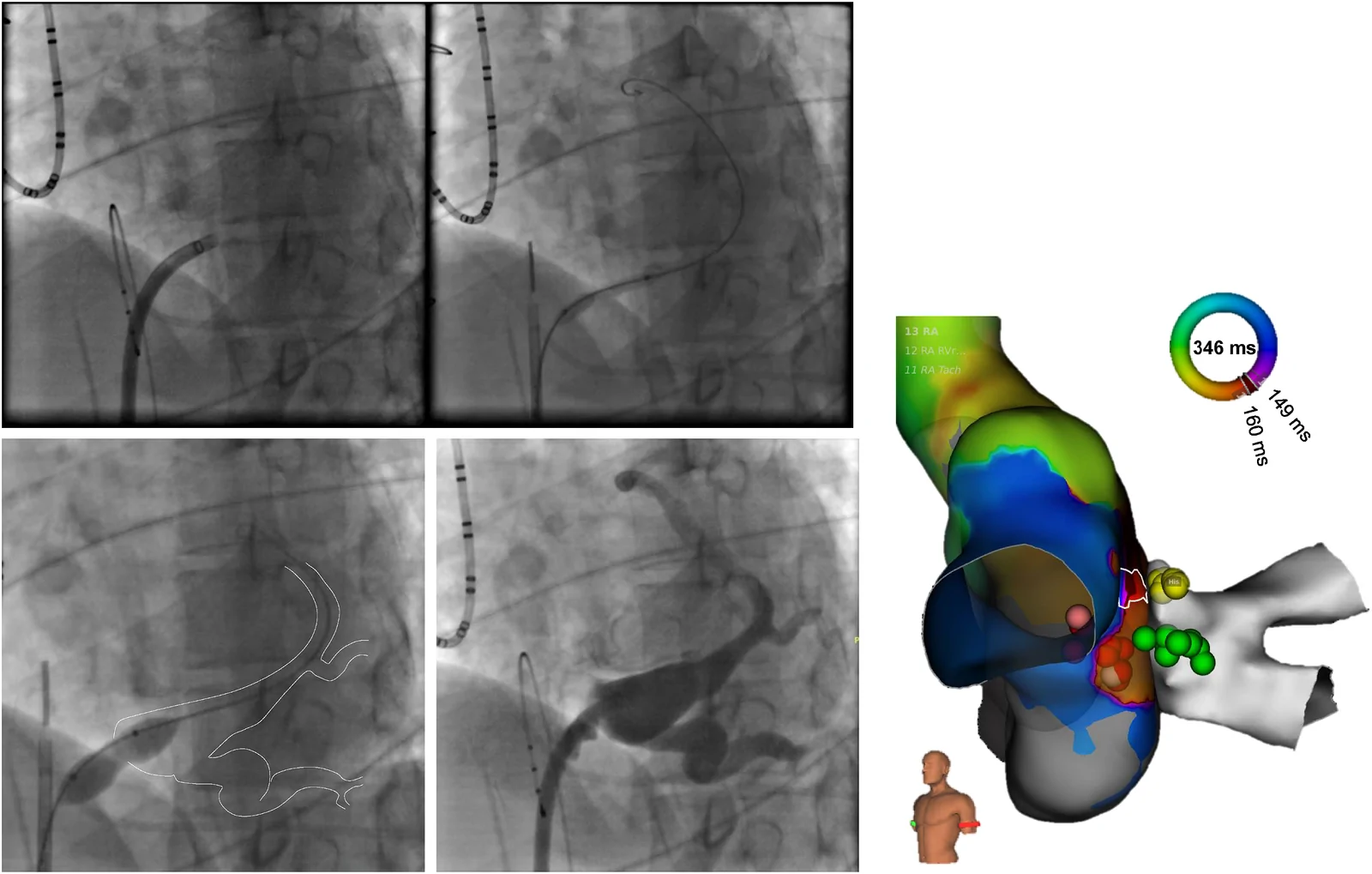
Example of coronary sinus patch occlusion related to prior operative repair of atrioventricular septal defect. This patient presented with recurrent supraventricular tachycardia. At the time of invasive electrophysiology study, AV node reentrant tachycardia (AVNRT) was induced. The coronary sinus, however, could not be cannulated and His bundle electrograms were not encountered. Catheter ablation required puncture into the obstructed coronary sinus. Near the inferiorly displaced AV node, radiofrequency lesions resulted in successful slow pathway modification and elimination of AVNRT.

Preparation begins with clear understanding of the patient's cardiac anatomy and the operative procedures that have been completed. It is important to review operative notes to ensure there is a good understanding of prior surgical procedures. A prime example is whether the coronary sinus drains to the right or the left atrial aspect, when pursuing CS catheter placement (Fig. 2). Review of cross-sectional imaging and 3D models of the patient's anatomy can be helpful to understand catheter courses and to plan for trans-septal or trans-baffle punctures when needed. Given the unusual location of AV conduction axis in CHD such as CCTGA, patient-specific anatomical considerations are important to prevent inadvertent AV nodal injury. Baffle leaks, if reported on prior imaging or catheter-based studies or discovered during concomitant transesophageal echocardiographic imaging, should be carefully assessed as these can potentially serve as an alternatives to trans-baffle punctures.

We routinely perform 3D segmentation to be integrated with the 3D mapping system for patients with congenital heart disease. Both CT and MRI with whole heart sequence can be used for this purpose. There are several platforms that can be used for such integration that allow segmentation. Annotating the AV valve annulus, coronary sinus and baffle structures can be quite helpful for mapping, accessing vessels and pursuing trans-baffle/trans-septal punctures.

### 2.4 Co-morbidities assessment

ACHD patients can present challenges in the management of co-morbidities. This might especially be the case for EP programs more accustomed to caring for pediatric and young adults without significant co-morbidities. A routine pre-operative assessment in conjunction with the ACHD team can be helpful. These could include routine risks such a management of peri-procedural anti-coagulation where standard guidelines apply. Special attention should be given to renal function when contrast use is anticipated. Respiratory compromise may dictate peri or post-operative care including a need for intensive care admission. The evaluation of coronary artery disease and hemodynamic assessment should be considered for possible combined hemodynamic catheter-based study along with the EP study.

### 2.5 Anesthesia considerations

We routinely perform the procedure under general anesthesia. Given the complexities and co-morbidities associated with ACHD patients, it is our routine practice to obtain a pre-procedural anesthesia consult. This allows pre-procedural planning and disposition post procedure. Propofol and inhaled anesthetics are typically used. In patients with a history of hemodynamically unstable tachycardia and those with ventricular dysfunction or other conditions that might place them at risk for hemodynamic compromise during the procedure, an arterial line for hemodynamic monitoring and a Foley catheter for more accurate volume assessment are placed. We typically prefer a radial arterial line in this situation which we find to minimize complications when compared to femoral arterial lines placed alongside large venous sheaths. A Foley catheter is considered for patients where protracted procedures are anticipated. Finally, in patients where ablation close to the phrenic nerve is anticipated with risk for diaphragmatic paralysis, a discussion with the anesthesiologist is useful to minimize use of paralytic agents or administration of reversible agents to check for phrenic nerve capture.

### 2.6 Access

**Fig. 3:**
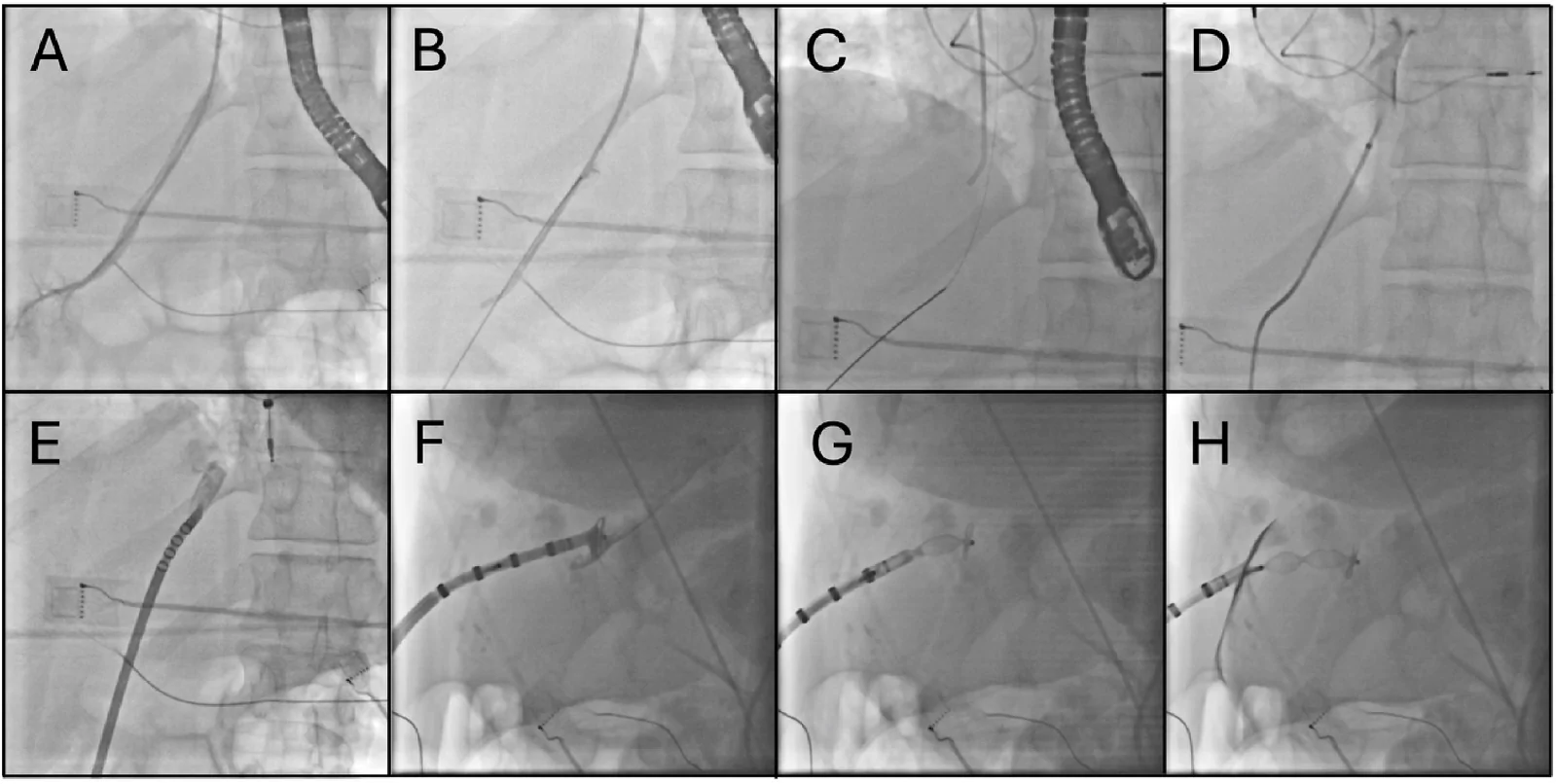
Transhepatic puncture for catheter ablation in congenital heart disease. Transhepatic puncture and sheath placement for a patient with left atrial isomerism and interrupted inferior vena cava who presented with symptomatic paroxysmal atrial fibrillation. Panels A shows a catheter that has been placed from the right internal jugular vein for contrast opacification of a hepatic vein branch. In panels B-E, needle puncture has been performed, followed by wire placement and successive catheter exchanges before placement of a deflectable mapping sheath. Following pulmonary vein isolation, contrast venography is used to position a vascular occlusion device to prevent hepatic bleeding (panels F-G).

In addition to reviewing the intra-cardiac anatomy an understanding of the patency of venous access is essential. It is not unusual for ACHD patients to have femoral vein occlusion or interruption of the infra-hepatic inferior vena cava, such that there is a need to utilize a superior approach or even trans-hepatic puncture (Fig. 3). In our institution pre-procedural ultrasound of the femoral veins is often performed for patients with concerns for femoral venous occlusion. A right internal jugular approach can often be helpful for accessing certain areas of the atrium and to access the coronary sinus if a complicated anatomy is anticipated.

Our standard setup includes, an 8Fr sheath for the mapping catheter, a 6 or 7 Fr sheath for a decapolar coronary sinus catheter and a 5Fr sheath for the Map-it His RV ™ catheter that allows recording and pacing at both the His bundle and the ventricle. To facilitate intra-cardiac echocardiogram (ICE), an additional 8 - 11 Fr sheath is placed depending upon the type of ICE catheter that is used. We prefer the pre-closure technique with the Abbott Perclose ™ system or post-procedure placement of a Haemonetics Vascade MVP® XL system, for hemostasis when using large sheath size especially in patients receiving anti-coagulation. Ultrasound is routinely used for improved access and minimizing complications such as inadvertent arterial puncture.

**Fig. 4:**
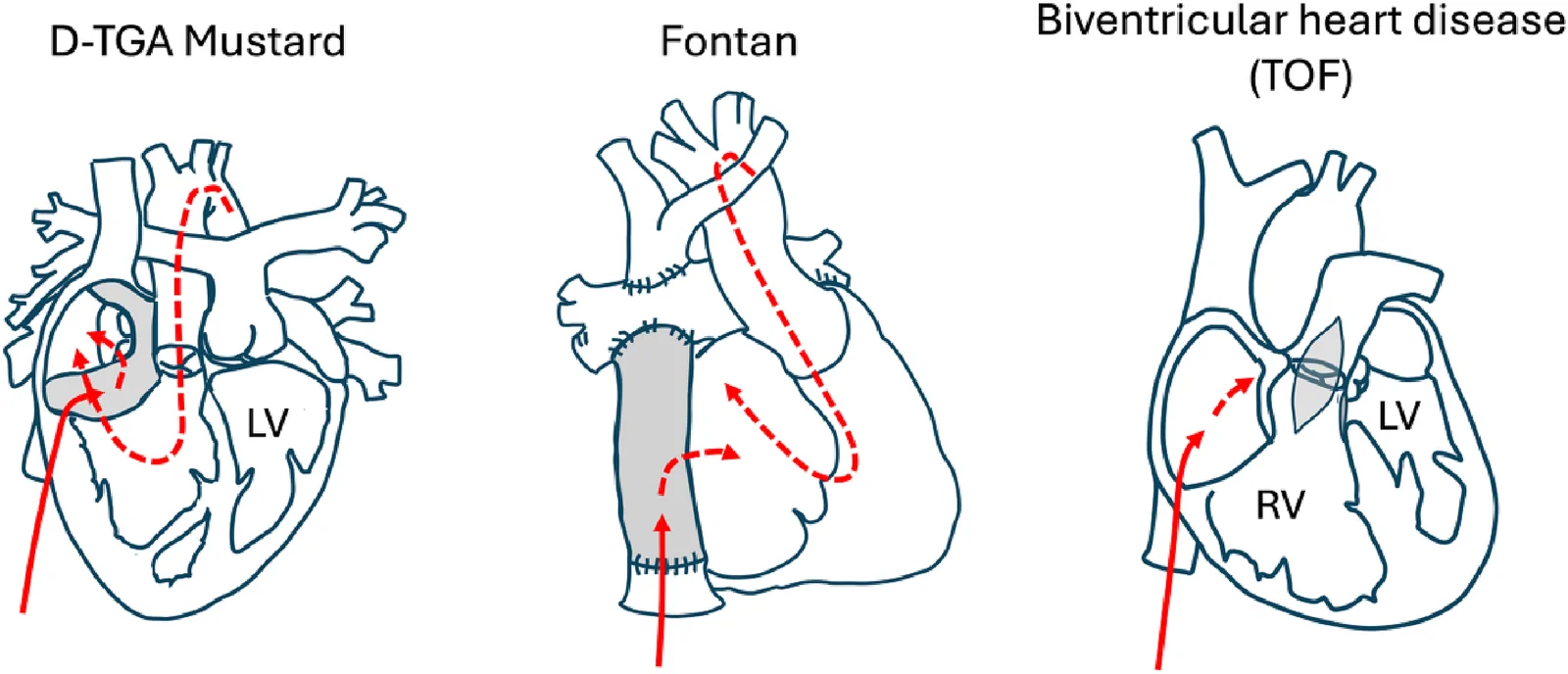
Cartoons illustrating cardiac access in complex congenital heart disease. The course of catheter placement (red arrows) for complex congenital heart disease is shown. For DTGA after the Mustard or Senning operation, puncture across the baffle is required. For Fontan patients, puncture through either a baffle or conduit may be necessary. For other defects, standard transseptal puncture may be pursued, but the operator may still contend with surgical ASD patch material. Although a retrograde approach may be used (dashed lines), catheter control is often suboptimal unless robotic magnetic navigation is also used.

Depending on the anatomical substrate, direct cardiac access can often be achieved using a standard approach from the inferior vena cava with standard transseptal puncture for left atrial arrhythmia substrates. However, for several complex ACHD anatomies, access to the cardiac chamber of interest may require catheter access across a baffle, surgical patch, or conduit (Fig. 4). This is particularly common after operative repair for d-transposition of the great arteries utilizing an atrial baffle operation and following repair for single ventricle anatomy, as with the Fontan operation. Electrophysiologists with extensive experience may learn to do these procedures independently or collaborate with interventional cardiologists to achieve access in these situations [3,4].

### 2.7 Intra-operative echocardiogram

We routinely perform ICE imaging prior to EP studies in ACHD patients. This allows clear delineation of the anatomy including structures such as the Eustachian valve and integration of the structures into the electrical mapping system. We have found ICE to be an additional tool to the use of segmented 3D image integration. We routinely mark the atrium, coronary sinus, structures such as the eustachian valve and the AV valve annulus on ICE imaging. In addition, transvenous leads can be marked when needed.

### 2.8 Mapping and ablation strategies

**Fig. 5:**
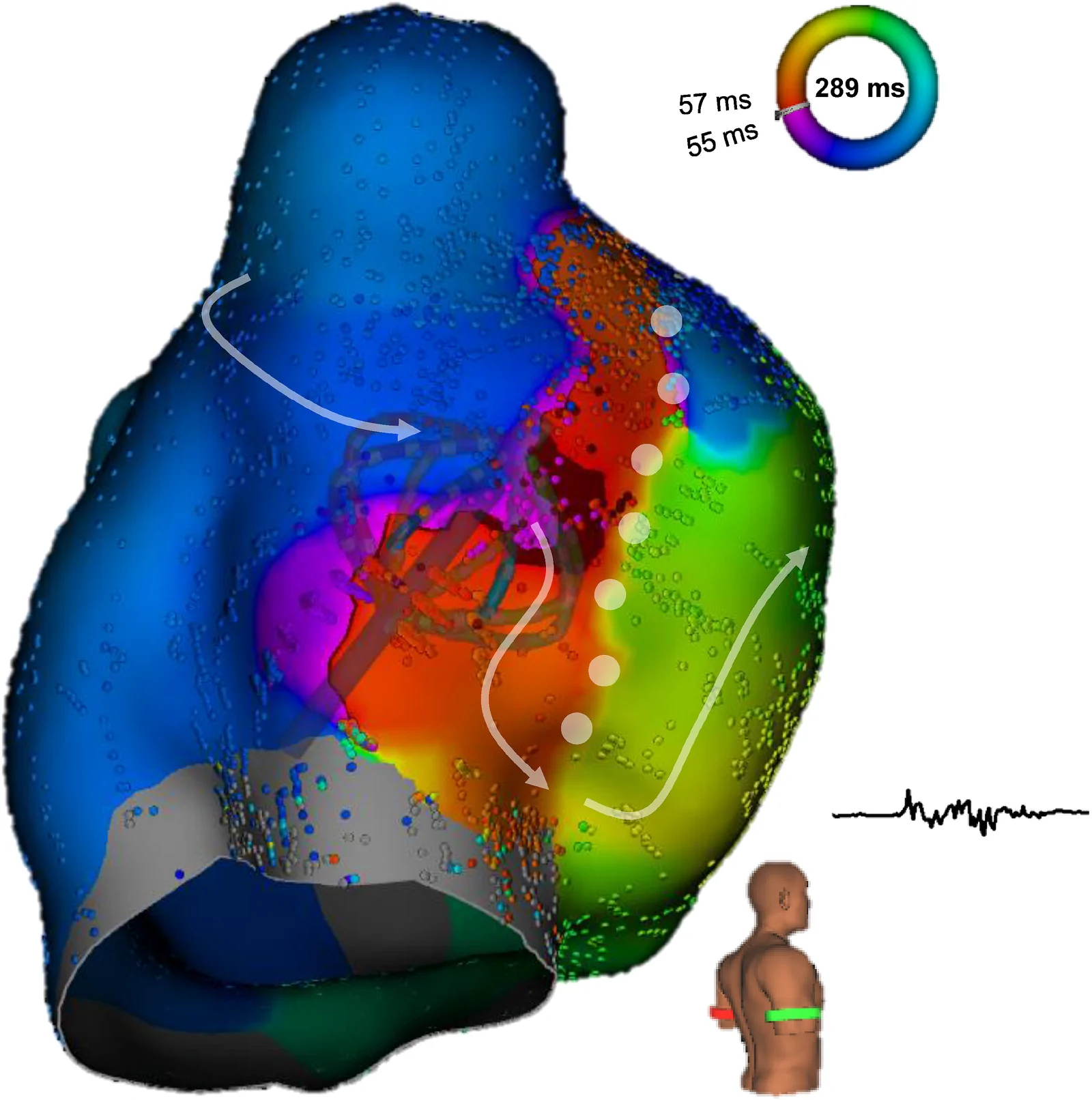
Example of tailored catheter ablation for adult congenital heart disease. With ultra-high-definition mapping, the wavefront associated with a macroreentrant tachycardia is seen to pass through separate conduction channels at the posterolateral right atrium. The first channel is associated with a narrow isthmus and highly fractionated electrograms, however risk for sinus node dysfunction is substantial here. A second inferior channel was therefore selected for catheter ablation which was remote from both the sinus node as well as the phrenic nerve to avoid procedural complication.

Following the creation of an anatomic blueprint using either or both the 3D image integration and ICE, we use a multi-electrode catheter. If we anticipate a significant scar burden such as a patient with Fontan palliation, our initial step is often to create a voltage and sinus activation map of the right sided structures. This allows one to define the anatomy well, define any pre-existing scars and have a good understanding of the location of the sinus node which might be pertinent (Fig. 5). We prefer to use a high-density multi-electrode mapping with close electrode spacing and low noise floor for mapping.

Following creation of a voltage map, we then move forward with a routine EPS with the goal of induction of tachycardia and understanding AV nodal conduction properties. Once a narrow or usual complex tachycardia is induced, our initial step is to first prove the mechanism of tachycardia in case of 1:1 tachycardia where other forms of SVT such as AVNRT/AVRT need to be excluded using standard maneuvers. Once an atrial tachycardia mechanism is apparent, we move forward with mapping the systemic venous atrium (SVA) with a multi-electrode high density catheter. A stable reference catheter is very important in this situation and having a CS catheter in the proper position can be valuable. In addition, an esophageal catheter can sometimes represent a stable reference electrode strategy. Activation map can quickly point towards micro/focal re-entrant tachycardia (mimicking a single focal point of origin - centrifugal activation) versus macro-re-entrant tachycardia. By evaluating wavefront propagation on the mapping platform, integration of vector and velocity information can lead to quick identification of functional scars and localization of the circuit. If the entire cycle length is unable to be mapped in a macro-re-entrant circuit, or if there is other evidence of circuit involving the pulmonary venous atrium (PVA), then a trans-septal or trans-baffle puncture is performed. Evidence of PVA involvement, can be often seen as early activation or diastolic potentials in the atrial septal region, on the CS or esophageal catheter. Using a steerable sheath can be helpful for mapping and for trans-septal/trans-baffle access. Once the macro-re-entrant tachycardia is localized, we judiciously utilize entrainment maneuvers for further confirmation if necessary.

**Fig. 6:**
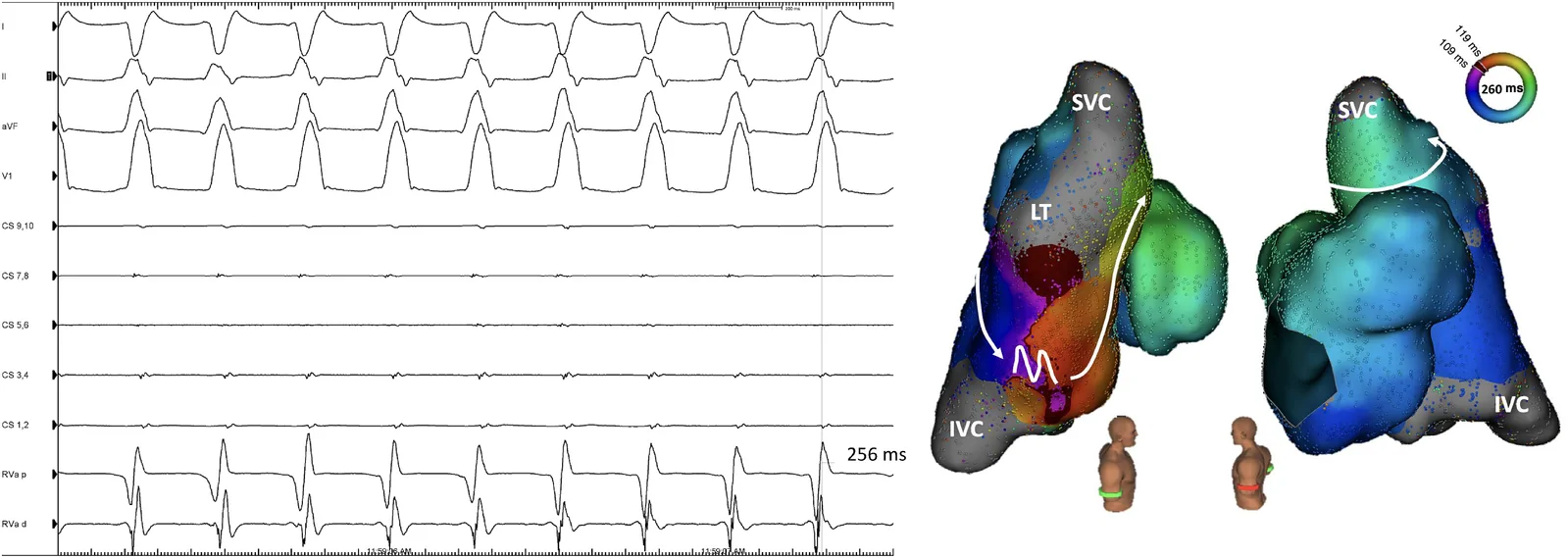
Rapid ventricular response with hemodynamic instability in congenital heart disease. An example of a patient with prior lateral tunnel Fontan operation and intra-atrial reentrant tachycardia (cycle length 260 ms) with 1:1 atrioventricular conduction during mapping is shown. The ensuring heart rate of 230 beats per minute resulted in rapid hemodynamic deterioration requiring cardioversion. Only after establishing adequate AV nodal blockade and optimizing anesthetic delivery could complete mapping be performed for successful catheter ablation.

Multiple tachycardia circuits can be challenging and often finding the dominant tachycardia, especially the one closest to the clinical tachycardia cycle length can be helpful. In hemodynamically unstable patients due to rapid 1:1 conduction, AV nodal blocking agents such as intra-venous calcium blockers can be helpful in slowing the ventricular response rate (Fig. 6).

Once the tachycardia circuit is identified, a lesion set is planned that bisects the shortest and safest anatomical isthmus. Important structures such as the AV/sinus node, coronary arteries, phrenic nerve and epicardial/transvenous leads should be taken into consideration when considering this lesion set. Phrenic nerve capture should be assessed by using high output pacing without paralytic effect prior to ablation. In regions close to the AV node, it is often prudent to perform the catheter ablation in sinus rhythm rather than during tachycardia to allow close monitoring of AV nodal conduction. We routinely attempt to check conduction time through the targeted isthmus prior to ablation. This can help facilitate understanding whether bi-directional block has been achieved following catheter ablation. Catheter ablation with irrigation and force sensing is ideal for patients with ACHD given an often thickened and hypertrophied atrial wall. Use of ICE during the ablation can often be helpful to further ensure contact and navigate complex structures such as the Eustachian valve. Targeting a far-field (generator) impedance drop of at least 10 Ω is ideal, although different mapping systems currently use multiple algorithms that can use products of contact force and temperature to determine effective lesions.

### 2.9 Procedural endpoints

In addition to non-inducibility of tachycardia with multiple sequences of atrial extra-stimuli, other endpoints should be documented in patients with macro-re-entrant atrial tachycardia. This includes proving bi-directional block across the ablation lesion set, by pacing on either side of the lesion set. This can also be accomplished by performing a continuous activation map of the peri-ablation site while pacing from each side consecutively, using a high-density multi-electrode catheter. In addition to confirming a clear line of block, this can demonstrate potential areas of residual isthmus conduction. In patients with multiple tachycardia circuits, the electrophysiologist may need to take into consideration the risk-benefit ratio of increasing rate of complications with prolonged anesthesia and risks of extensive ablation when compared to the potential benefit. In some patients, consideration for a pacemaker with ATP options or atrial pacing to allow higher doses of anti-arrhythmic medications may need to be considered.

## 3 Future directions

Catheter ablation in patients with congenital heart disease benefits from a greater understanding of the unique tachycardia mechanisms, along with significant improvement in mapping and ablation technologies over the past decade. Accordingly, novel technologies such as pulsed field ablation may add to the armamentarium available to the congenital electrophysiologists when confronted with substrates that are historically less response to radiofrequency energy (e.g. densely fibrotic areas or surrounding by prosthetic material). Future research should explore the utility of this and other ablation technologies. In addition, the evolution from atrial macro reentry to AF over the lifespan is now apparent and our understanding of the complex interplay between these tachycardia mechanisms is evolving. Future research should focus on the optimal strategy for prevention and management of the emerging AF epidemic.

## Declaration of competing interest

No relevant disclosures. The author received no funding related to this work.
